# Widespread coral bleaching and mass mortality during the 2023–2024 marine heatwave in Little Cayman

**DOI:** 10.1371/journal.pone.0322636

**Published:** 2025-05-02

**Authors:** Matthew L. Doherty, Jack V. Johnson, Gretchen Goodbody-Gringley

**Affiliations:** 1 School of Biological and Marine Sciences, University of Plymouth, Plymouth, United Kingdom; 2 Reef Ecology and Evolution Lab, Central Caribbean Marine Institute, Little Cayman Island, Cayman Islands; University of the Ryukyus, JAPAN

## Abstract

The increased frequency and intensity of marine heatwaves (MHWs) induced by continued global warming are the greatest threat to tropical coral reefs, causing mass bleaching events and widespread mortality of reef building corals. In 2023, the isolated and well-protected reefs around Little Cayman experienced a MHW of > 17 Degree Heating Weeks (DHW), far exceeding any DHW measure previously captured. During the peak of the heatwave, ~ 80% of all corals were either bleached or showing signs of mortality. On the final survey date ~54% of all corals surveyed were recorded as dead. However, we identified significant differences in bleaching susceptibility and mortality across taxonomic groups, related to different life history strategies. Notably, weedy coral taxa such as *Agaricia spp., Porites astreoides, and Porites porites,* experienced high bleaching and suffered extensive mortality. Meanwhile, stress-tolerant reef building taxa such as *Orbicella spp*., experienced bleaching, but suffered low mortality. Given Little Cayman reefs have not been exposed to previous thermal stress events, the highly sensitive weedy taxa disproportionately contributed to coral abundance. Thus, the occurrence of a high magnitude – long duration heatwave resulted in catastrophic mortality of corals in Little Cayman, despite ~57% of the coastal environment being classified as no-take Marine Protected Areas. These findings underscore that the global stressor of global climate change, which drives MHWs, cannot be mitigated by local protection and isolation, thus highlighting the need to directly tackle the cause of coral decline (i.e., global climate change).

## Introduction

Since 1955, the oceans have absorbed approximately 93% of the excess heat generated by greenhouse gas emissions, which has significantly impacted marine environments [1,2]. This warming has exacerbated the frequency and intensity of El Niño-Southern Oscillation (ENSO) events contributing to an increased intensity and frequency of marine heatwaves (MHWs) [3]. These events are characterised by warm sea surface temperatures that last from days to months and can extend over a large spatial range [4]. In 2023, global temperatures were 1.48 °C higher compared to the 20^th^ century average as a result of continued global warming and ENSO events [5].

Over the past 30 years, average coral cover on tropical reefs worldwide has declined from ~60% to < 20% [6–14]. While there are numerous local causes of coral loss (e.g., pollution, destructive fishing practices, tourism, etc.), the single most detrimental stressor to date is thermal stress from anomalous heating events (i.e., heatwaves) and its associated complications (i.e., bleaching, disease, reduced calcification etc.; [12,15,16]. Scleractinian corals live close to their thermal maximum, and thus even slight increases in temperatures can lead to the loss of autotrophic symbionts (dysbiosis) in a process known as coral bleaching [17–19]. Prolonged periods of bleaching may ultimately lead to coral mortality. Many species are reliant on translocated photosynthate from endosymbiotic dinoflagellates in the family Symbiodiniaceae to meet the majority of their metabolic demands [20]. As global temperatures continue to rise, so too does the frequency, intensity, and duration of coral bleaching [12,21].

The first widespread bleaching event was documented during the 1982–83 ENSO [22,23], with the first global bleaching event occurring in 1998 impacting coral cover across all tropical locations [24–26]. A subsequent global bleaching event occurred in 2010. Another ENSO driven event occurred just four year later, spanning 2014–2016, and became the most severe and widespread bleaching event on record [27]. In the Spring of 2024 as the impacts of the El Nino event began to impact the Southern Hemisphere, the fourth Global Bleaching Event was announced [28].

While previous global bleaching events have impacted the Cayman Islands, long term monitoring data from Little Cayman Island indicated sustained coral cover of roughly 22% between 1999–2019 [29,30]. This stability suggests these reefs were less impacted than others across the region, which was suggested to be in response to strong local protection measures and low human population. However, in 2023, sea surface temperatures in Little Cayman exceeded all previous records, reaching an unprecedented 17.55 Degree Heating Week. In response to these increasing temperatures, we began a series of surveys across several sites around Little Cayman, where roughly 57% of the nearshore environment is under full no-take protection. This provided a rare opportunity to assess bleaching dynamics and associated coral mortality within an ecosystem largely free from local anthropogenic stressors.

## Method

### Study site

**Fig 1:**
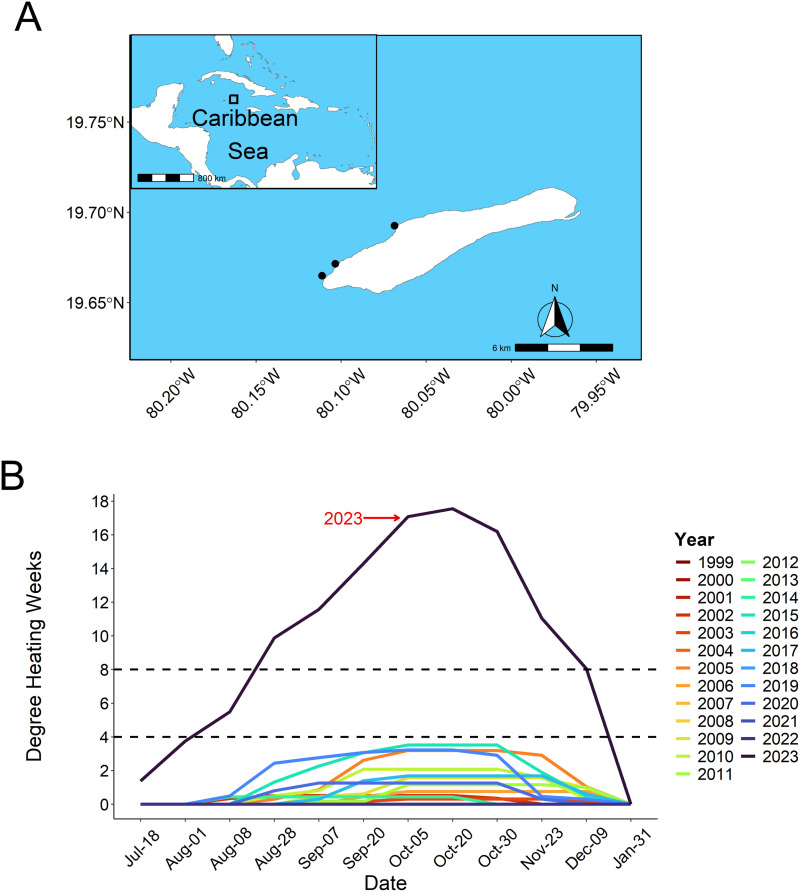
(A) Location of the three survey sites in Little Cayman, situated within the Caribbean sea, using maps generated with the rnaturalearth package [32]. Survey sites from East to West are Meadows (19.69195° N, -80.06870° W), Jigsaw (19.671472° N, -80.103389° W), and Martha’s Finyard (19.68677° N, -80.05860° W). (**B**) shows Little Caymans thermal history from 1999–2023 based on degree heating weeks (DHW) for every year during the same survey as 2023. DHW values were obtained from the NOAA Coral Reef Watch 5km product suite [33], using Jigsaw as the reference site. Dashed vertical lines represent 4 and 8 DHW, typical predictors of widespread bleaching, and bleaching induced mortality.

The Cayman Islands are located 200 miles northwest of Jamaica and 150 miles south of Cuba in the Caribbean Sea and are comprised of three main islands: Grand Cayman, Cayman Brac, and Little Cayman. Little Cayman (Fig 1A), located 80 miles northeast of Grand Cayman, is the smallest and least developed of the three islands with an area of roughly 26km^2^ and a population of 160 permanent residents. Of the 45 km of shoreline, 74.2% is designated as marine protected areas with roughly 57% under full No-Take protection by the Cayman Islands government [31].

The island is surrounded by a fringing reef system that transitions from spur and groove formations to a fore reef wall, which exceeds 2000m depth. Three sites were surveyed on the north side of Little Cayman; Martha’s Finyard (15m depth), Jigsaw (15m depth), and Meadows (6m depth), arranged from east to west respectively (Fig 1A). All sites feature coral assemblages dominated by weedy taxa such as *Agaricia spp.* and *Porites astreoides* and experience similar seasonal variations in temperature and light with periodic storm surges throughout the year. All surveys were conducted under blanket permits for non-extractive coral research, issued by the Cayman Islands Department of Environment. No corals were collected, removed, or manipulated during the study.

### Temperature stress accumulation

To predict mass bleaching events, Degree Heating Weeks (DHW) can be used as a key global indicator, with one DHW quantified as a sustained increase of 1°C above the long-term average temperature for the warmest month in a specific area [34]. The coordinates for Little Cayman were specified to the site Jigsaw (19.671480, -80.103380), selected as the central East to West study site. For each date in this study, as well as historical data, DHWs were extracted from NOAA’s Coral Reef Watch 5km product suite ([33]; Fig 1B) using the above co-ordinates.

### Survey methods

At each site, a single 30m transect was surveyed using the coral health monitoring protocol as described in [35]. Surveys were repeated bi-weekly (weather dependent), from July 18, 2023, to Jan 31, 2024, to ensure capture of peak bleaching (the timepoint at which the number of bleached colonies surveyed was at its highest), peak mortality (the timepoint at the number of recently dead colonies surveyed was at its highest), as well as recovery. To facilitate repeated surveys without permanent markers, transects were laid from a set starting point and followed the same compass heading at each subsequent time point. Surge and currents naturally moved the transects, causing fluctuations in exact positioning of transects between surveys and thus the same individual colonies were not necessarily assessed on each survey. As such, mortality rates were assessed as cumulative over the course of the survey period, with the total number of recently dead colonies increasing throughout the study.

During each survey, corals within 1m to the right of the transect from the starting point were identified to species level. Exceptions to this were *Agaricia spp*, *Scolymia spp*, and *Mycetophyllia spp*, which were identified to genus due to the challenges of accurate species level identification without genetic analysis. Colony size was measured as maximum height and maximum diameter and put into the following size classes (cm), based on the maximum measurement between either category: 1–4 (juvenile), 1–4 (isolate), 5–10, 11–20, 21–40, 41–80, 80 + . Juveniles are defined as coral recruits larger than 1 cm, while isolates refer to remnants of older colonies which persist after partial mortality.

### Colony health assessment

Colony health was assessed and categorised into one or more of the following conditions: colour loss (paling or bleaching), discoloration, growth anomalies, tissue loss (percentage and pattern of lesions noted), partial mortality (visually estimated with a percentage), and total mortality, as described by Harper [35]. Paling refers to a reduction in pigmentation due to a decrease in Symbiodiniaceae, while bleaching refers to a complete loss in Symbiodiniaceae resulting in a stark white appearance with living tissue covering the skeleton. Discolouration refers to abnormal pigmentation that does not fall under paling or bleaching, such as darkening. Growth anomalies are abnormal skeletal formations which appear as tumour like morphotypes on coral tissue. Tissue loss refers to the absence of live coral tissue, exposing fresh skeleton, which may be surrounded by healthy coral, or a progressing band of affected tissue. Mortality was identified by the complete absence of living tissue, leaving behind bare skeleton that is often overgrown by algae.

### Statistical analysis

Data handling and analyses were conducted using R 4.4.1 [36]. To statistically assess changes in coral health throughout the survey period, Generalised Additive Models (GAMs) were used for each health category, with time (survey period) as the predictor. The four GAMs were specified with a beta distribution as % of each health condition represent proportional data, with p-value corrections using the conservative Bonferonni adjustment. Models included the random effect of site to account for site-scale variation in health conditions. GAMs were constructed using the ‘MGCV’ package [37]. Generalised Linear Models (GLMs) specified with a beta distribution were used to statistically assess the influence of DHW on proportion of coral bleaching and coral health, constructed with the ‘betareg’ package [38]. A GLM was also used to assess the change in coral abundance over the survey period, specified with a Poisson distribution as these data are counts. Comparisons between species for bleaching and mortality were statistically compared using a Kruskal-Wallis test given the data did not meet assumptions of a normal distribution. Post-hoc pairwise comparisons were made using a Dunns test from the FSA package [39]. P-values were not adjusted post-hoc as we only had 3 replicates at the time for mean maximum bleaching providing insufficient statistical power for p-value corrections. A Mann-Whitney U test was used to compare bleaching and mortality between different coral life history strategies.

## Results

**Fig 2:**
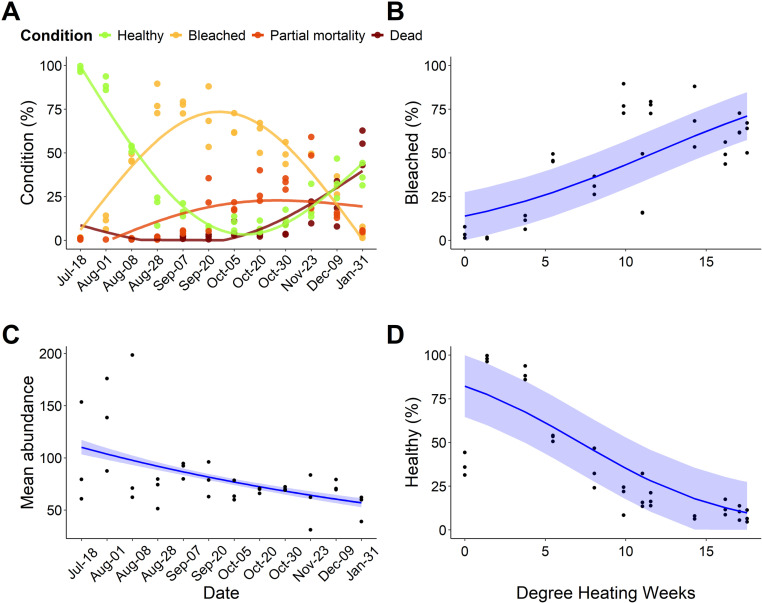
(A) coral health conditions throughout the survey period. Points represent the overall percentage of corals in each condition category for each site. Lines depict the non-linear trend based on a Generalized Additive Model. (B) The percent of corals bleached at each site in response to Degree Heating Weeks (DHW). Linear trend fitted with a beta regression, with ribbons showing the standard error (SE). (C) Coral abundance at each site throughout the survey period. Linear trend from a Generalized Linear Model parameter with a Poisson family distribution. Ribbons show 95% confidence intervals. (D) Percent of healthy corals in relation to DHW throughout the survey period, with linear trend from a beta regression, and ribbons showing SE.

Coral bleaching impacts peaked on the 20th of September 2023 with only 7.38% ± 0.57 (SE) % of all corals remaining healthy, meaning 92.62% of all corals were either bleached, or experiencing mortality. Whole colony mortality of corals peaked after the marine heatwave had subsided on the 31^st^ of January 2024, with 53.65 ± 5.77 (SE) % of coral colonies on our original transects dead. Healthy corals were at their lowest frequency during the end of September through to the end of October 2023, coinciding with the peak of thermal stress. The mean lowest percent of healthy corals occurred on the 20^th^ of September 2023, with only 7.38 ± 0.57 (SE) % of corals not experiencing either bleaching or mortality (Fig 2A). Coral bleaching significantly increased as thermal stress (DHW) increased (GLM, estimate = 1.55, Z = 5.551, p < 0.001, Fig 2B). Mean abundance of corals significantly declined during the survey period (estimate = -0.2, Z = 11.06, p < 0.001, Fig 2C), while the proportion of healthy corals also significantly decreased as DHW increased (estimate = -0.214, Z = -7.3, p < 0.001, Fig 2D).

### Species specific bleaching and mortality

**Fig 3:**
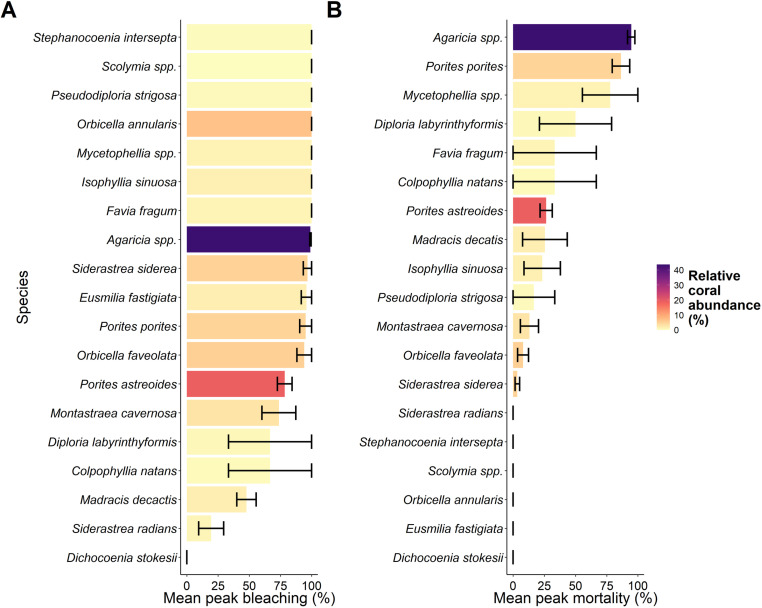
Species specific maximum bleaching (the timepoint at which the mean bleaching percentage was at its highest) (A), and maximum mortality (the timepoint at the mean mortality percentage was at its highest) (B). Mean was calculated from the maximum percent at the three sites, with error bars showing standard error. Colours display the relative coral abundance (%) from surveys.

Coral bleaching (Kruskal = Wallis, χ^2^ = 37.606, df = 18, p = 0.004) and coral mortality (χ^2^ = 33.868, df = 18, p = 0.013) varied significantly between species (Fig 3). A full breakdown of pairwise comparisons for species are available for coral bleaching in S2 Table in S1 File, and for mortality in S3 Table in S1 File.

Several coral species experienced 100% coral bleaching, including the critical Caribbean reef building species *Orbicella annualris.* From the most abundant corals in Little Cayman, making up 43.56% of all corals surveyed, *Agaricia spp*., experienced 98.98±0.59 (SE) % bleaching leading to 94.65 ± 2.92 (SE) % mortality by the end of the survey period. The second most abundant species, *P. astreoides* experienced 78.44 ± 5.89 (SE) % bleaching, but in contrast only suffered 26.5 ± 4.86 (SE) % mortality. All coral species/genera surveyed across these transects experienced bleaching, except for *Dichocoenia stokesii,* and 13 out of 19 suffered some level of mortality (Table 1).

**Table 1:** Species specific responses to 2023 heat wave. Frequency (%) indicates the percent contribution of each species to overall coral composition; Bleaching (%) represents the peak percent bleaching; Date is the date on which peak bleaching occurred; DHW indicates the degree heating week at peak bleaching; Under the mortality header, Date indicates the date on which peak mortality occurred while Mortality (%) represents the percent of entire coral colonies that were dead by the end of the survey period.

AGRRA code	Species	Bleaching				Mortality	
		Frequency (%)	Bleaching (%)	Date	DHW	Date	Mortality (%)
agga	*Agaricia spp.*	43.56	98.98	28-Aug-23	9.87	31-Jan-24	94.65
cnat	*Colpophyllia natans*	0.50	66.67	20-Sep-23	14.29	31-Jan-24	33.33
dlab	*Diploria labyrinthiformis*	0.76	66.67	01-Aug-23	3.75	01-Aug-23	50.00
dsto	*Dichocoenia stokesii*	0.24	0.00	01-Aug-23	3.75	01-Aug-23	0.00
efas	*Eusmilia fastigiata*	1.82	95.83	23-Nov-23	11.04	31-Jan-24	0.00
ffra	*Favia fragum*	1.17	100.00	01-Aug-23	3.75	01-Aug-23	33.33
isin	*Isophyllia sinuosa*	1.67	100.00	05-Oct-23	17.08	31-Jan-24	23.33
mcav	*Montastraea cavernosa*	3.02	73.74	20-Oct-23	17.55	20-Sep-23	13.10
mdec	*Madracis decactis*	1.85	47.78	20-Sep-23	14.29	20-Sep-23	25.56
myce	*Mycetophyllia spp.*	1.41	100.00	20-Sep-23	14.29	07-Sep-23	77.78
oann	*Orbicella annularis*	6.85	100.00	28-Aug-23	9.87	31-Jan-24	0.00
ofav	*Orbicella faveolata*	5.35	94.12	28-Aug-23	9.87	31-Jan-24	8.03
past	*Porites astreoides*	18.78	78.44	28-Aug-23	9.87	05-Oct-23	26.50
ppor	*Porites porites*	4.92	95.24	28-Aug-23	9.87	31-Jan-24	86.43
pstr	*Pseudodiploria strigosa*	0.51	100.00	09-Dec-23	8.05	18-Jul-23	16.67
scol	*Scolymia spp.*	0.10	100.00	09-Dec-23	8.05	01-Aug-23	0.00
sint	*Stephanocoenia intersepta*	0.30	100.00	23-Nov-23	11.04	01-Aug-23	0.00
srad	*Siderastrea radians*	1.12	19.44	23-Nov-23	11.04	31-Aus-23	0.00
ssid	*Siderastrea siderea*	5.03	96.67	28-Aug-23	9.87	08-Aus-23	3.42

### Coral life history strategy

**Fig 4:**
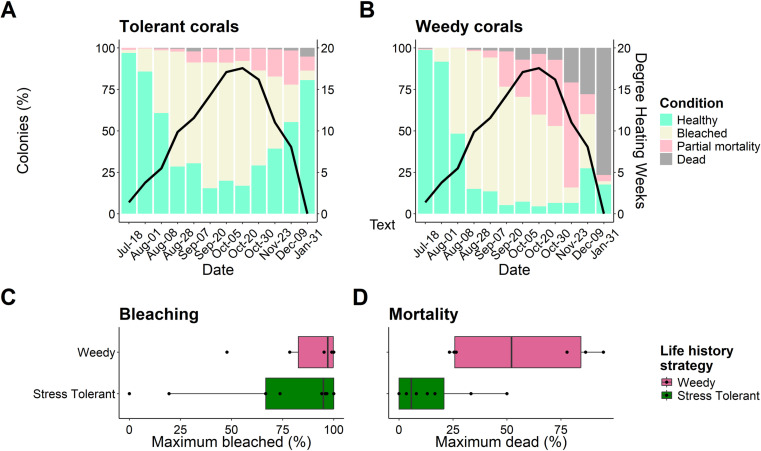
Species specific relationship between maximum bleaching and maximum mortality in Little Cayman. Points with labels show the species codes, with the full list of codes and their corresponding species in Table 1. Colours represent the species growth form. The dashed vertical line depicts the mean maximum bleaching for species, while the dashed horizontal line shows the mean maximum mortality of coral species.

No significant relationship existed between the mean maximum percent mortality and mean maximum percent bleaching (Fig 4). This was consistent overall, and for both weedy and stress tolerant coral species. Weedy coral species generally experienced high bleaching and high mortality, while stress tolerant species experienced high bleaching but high survival (Fig 4).

**Fig 5:**
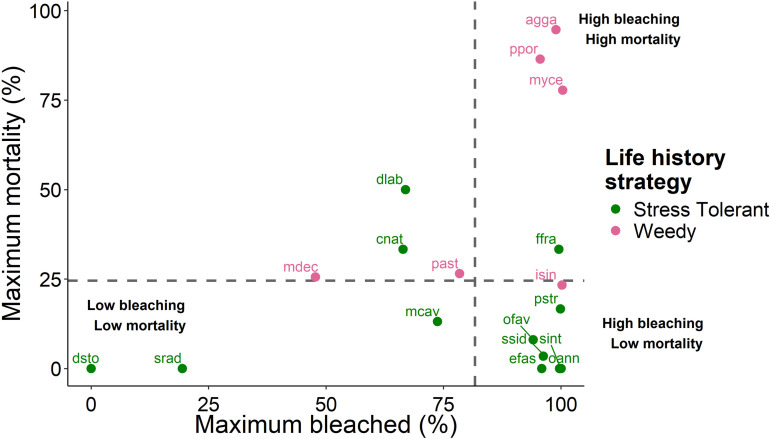
Comparisons of health conditions for (A) tolerant coral species, and (B) weedy coral species. Black solid line depicts degree heating weeks throughout the survey period. (**C**) shows compares the maximum bleaching (%) between weedy and tolerant coral species, while (**D**) shows maximum mortality (%). Points represent species specific values, the boxes are the 1^st^ (Q1) and 3^rd^ (Q3) interquartile range, black vertical bar is the median, while whiskers show the maximum values calculated at 1.5 x the interquartile range.

Mean maximum bleaching did not differ based on life history strategy. However, mortality was significantly higher for weedy corals compared to stress-tolerant corals (Man-Whitney U, W = 9, p = 0.012, Fig 5).

## Discussion

The reef system in Little Cayman is well documented to exhibit relatively high coral cover compared to the Caribbean that has been stable at roughly 22–24% since recurrent surveying began in 1999 [30]. This stability is often attributed to the high degree of marine protection in Little Cayman, which encompasses 74.2% of the shoreline, which is well above the United Nations suggested 30% by 2030 [40]. However, local protection and high coral cover do not provide refuge to the extreme heat waves caused by human induced climate change [41–43]. Our results document catastrophic coral loss with ~80% of corals bleached at the peak of the event and ~ 54% experiencing whole colony mortality at the end of the study.

Reef-building corals live close to their physiological thermal maximum, and as a result, warming of 1°C or more above local mean monthly maxima can cause bleaching, tissue loss, or whole-colony mortality [44,45]. The likelihood of coral bleaching increases with rising temperature stress, yet this response depends both on the scale and duration of the heating event [46–48], as well as the thermal history of the location [49–51]. In fact, several studies have found that previous experiences can shape the susceptibility of corals to bleaching (e.g., [52]). For example, a history of high temperature variability was found to decrease bleaching susceptibility [21,50,53], while repetitive high intensity and high frequency temperature stress events result in severe bleaching [21,54]. Coral bleaching has occurred in Little Cayman previously [55], yet mortality did not ensue, and the thermal stress was far below the 4DHW threshold when using contemporary satellite data (Fig 1B). Prior to 2023, Little Cayman had never experienced an event exceeding 4 DHW, with the second strongest event occurring in 2015 at 3.52 DHW [56]. Stress events exceeding 8 DHW is when mass bleaching is expected to occur [57,58], making the 17.55 DHW experienced in 2023 extreme by comparison. Thus, the lack of historical exposure to extreme heat waves may have left the reefs of Little Cayman stable with high coral cover and abundance, yet less resilient to bleaching impacts [52].

As expected, the bleaching event in Little Cayman had significant impacts that differed among taxonomic groups (genera and species). For example, slow growing, stress-tolerant reef-building taxa (e.g., *Orbicella spp*.) experienced extensive bleaching, but low mortality, suggesting high potential to recover from thermal stress. Such resilience of key reef building taxa provides positive implications for persistence of coral reefs through continued global warming. Yet, taxa highly susceptible to thermally induced bleaching such as *Agaricia spp., Porites porites,* and *Mycetophyllia spp.* showed extremely high levels of bleaching (>95%) and high mortality. Given these taxa are often fast growing, and prolific recruiters, they disproportionately contribute to the raw abundance of corals throughout Little Cayman’s coral reefs (see Fig 3). Thus, the occurrence of what was previously an unprecedented MHW of over 17 DHW in Little Cayman resulted in catastrophic levels of coral mortality. As such, it is likely the coral community composition of Little Cayman may be permanently altered by this bleaching event as the oceans continue to warm [59], likely transitioning into low coral cover states dominated by slow growing stress tolerant coral taxa rather than highly abundant weedy species [60].

Overall, the 2023 MHW has profoundly impacted the coral reef communities of Little Cayman, marking a significant ecological shift. Given the historical data from similar events in other Caribbean locations, the prospects for recovery of these reefs appear grim. Previously recognized by UNESCO as among the healthiest in the Caribbean [40], Little Cayman reefs are now likely to mirror the degraded systems observed across the region (e.g.,: [61–63]). Importantly, the extensive marine protected areas that encompass nearly 75% of the nearshore environment were unable to buffer these reefs from this extreme MHW. While several studies have touted local protection, limited development, and reduced human population as mechanisms to increase reef resilience (i.e., [64,65]), the results reported here suggest that no degree of local protection can safeguard corals from the future increases in seawater temperature expected with global climate change.

## Supporting information

S1 File Supplementary Figures and Tables. Includes **S1 Table** (GAM model outputs for coral conditions**), S2 Table** (pairwise comparisons of bleaching severity), **S3 Table** (pairwise comparisons of mortality), and **S1 Fig** (overview of coral conditions and Degree Heating Weeks).
(DOCX)
