# Supplementary material for: Widespread coral bleaching and mass mortality during the 2023–2024 marine heatwave in Little Cayman
Source: PLoS One. 2025 May 2;20(5):e0322636. doi: 10.1371/journal.pone.0322636 (PMC12047782; doi:10.1371/journal.pone.0322636)
Supplement: S1 File — Includes S1 Table (GAM model outputs for coral conditions), S2 Table (pairwise comparisons of bleaching severity), S3 Table (pairwise comparisons of mortality), and S1 Fig (overview of coral conditions and Degree Heating Weeks). (DOCX) [file pone.0322636.s001.docx]

Supplementary information for:

**Widespread coral bleaching and mass mortality during the 2023 marine heatwave in Little Cayman**

Doherty, Matthew L^1^, Johnson, Jack V^2^, Goodbody-Gringley, Gretche^2^

^1^ School of Biological and Marine Sciences, University of Plymouth, Plymouth, United Kingdom

^2^ Central Caribbean Marine Institute, Reef Ecology and Evolution, Little Cayman Island, Cayman Islands

* Corresponding author

E-mail: dohertym@protonmail.com

**S1 Table.** Outputs from Generalized Additive Models for each coral condition with time (sampling period) as the predictor, and % of the specified coral condition as the response variable. The heatwave (i.e. time) explained a large amount of deviance for coral conditions. (.

| **Coral condition** | **edf** | **Ref.df** | **Chi.sq** | **p-value** | **R-sq. (adj)** | **Deviance explained** | **n** |
| --- | --- | --- | --- | --- | --- | --- | --- |
| **Bleached** | 5.922 | 7.122 | 173.228 | <0.001 | 0.889 | 92.9 | 35 |
| **Dead** | 3.58 | 4.424 | 189.25 | <0.001 | 0.901 | 90.9 | 27 |
| **Healthy** | 5.675 | 6.838 | 353.3 | <0.001 | 0.962 | 97.2 | 36 |
| **Partial mortality** | 4.84 | 5.886 | 104.9 | <0.001 | 0.682 | 87.5 | 33 |

**S2 Table.** Pairwise comparisons of maximum bleaching severity between species. Only significant values are displayed in the table. All other comparisons were non-significant. P-values were calculated from a DunnsTest using no correction owing to low statistical power (n=3). AGRRA code genus and species names are available in Table 1.

| **Comparison (AGRRA code)** | **Z** | **P.unadj** |
| --- | --- | --- |
| agga - dsto | 2.036 | 0.042 |
| cnat - dsto | 2.050 | 0.040 |
| dlab - dsto | 2.050 | 0.040 |
| dsto - efas | -2.457 | 0.014 |
| dsto - ffra | -3.074 | 0.002 |
| dsto - isin | -3.074 | 0.002 |
| ffra - mdec | 2.513 | 0.012 |
| isin - mdec | 2.513 | 0.012 |
| dsto - myce | -3.074 | 0.002 |
| mdec - myce | -2.513 | 0.012 |
| dsto - oann | -3.074 | 0.002 |
| mdec - oann | -2.513 | 0.012 |
| dsto - ofav | -2.401 | 0.016 |
| ffra - past | 2.022 | 0.043 |
| isin - past | 2.022 | 0.043 |
| myce - past | 2.022 | 0.043 |
| oann - past | 2.022 | 0.043 |
| dsto - ppor | -2.429 | 0.015 |
| dsto - pstr | -3.074 | 0.002 |
| mdec - pstr | -2.513 | 0.012 |
| past - pstr | -2.022 | 0.043 |
| dsto - scol | -3.074 | 0.002 |
| mdec - scol | -2.513 | 0.012 |
| past - scol | -2.022 | 0.043 |
| dsto - sint | -3.074 | 0.002 |
| mdec - sint | -2.513 | 0.012 |
| past - sint | -2.022 | 0.043 |
| efas - srad | 2.218 | 0.027 |
| ffra - srad | 2.836 | 0.005 |
| isin - srad | 2.836 | 0.005 |
| myce - srad | 2.836 | 0.005 |
| oann - srad | 2.836 | 0.005 |
| ofav - srad | 2.162 | 0.031 |
| ppor - srad | 2.190 | 0.029 |
| pstr - srad | 2.836 | 0.005 |
| scol - srad | 2.836 | 0.005 |
| sint - srad | 2.836 | 0.005 |
| dsto - ssid | -2.513 | 0.012 |
| srad - ssid | -2.274 | 0.023 |

**S3 Table.** Pairwise comparisons of maximum mortality between species. Only significant values are displayed in the table. All other comparisons were non-significant. P-values were calculated from a DunnsTest using no correction owing to low statistical power (n=3). AGRRA code genus and species names are available in Table 1.

| **Comparison (AGRRA code)** | **Z** | **P.unadj** |
| --- | --- | --- |
| agga - dsto | 2.756 | 0.006 |
| agga - efas | 2.756 | 0.006 |
| dsto - myce | -2.756 | 0.006 |
| efas - myce | -2.756 | 0.006 |
| agga - oann | 2.756 | 0.006 |
| myce - oann | 2.756 | 0.006 |
| dsto - ppor | -2.743 | 0.006 |
| efas - ppor | -2.743 | 0.006 |
| oann - ppor | -2.743 | 0.006 |
| agga - pstr | 1.997 | 0.046 |
| myce - pstr | 1.997 | 0.046 |
| ppor - pstr | 1.984 | 0.047 |
| agga - scol | 2.756 | 0.006 |
| myce - scol | 2.756 | 0.006 |
| ppor - scol | 2.743 | 0.006 |
| agga - sint | 2.756 | 0.006 |
| myce - sint | 2.756 | 0.006 |
| ppor - sint | 2.743 | 0.006 |
| agga - srad | 2.756 | 0.006 |
| myce - srad | 2.756 | 0.006 |
| ppor - srad | 2.743 | 0.006 |


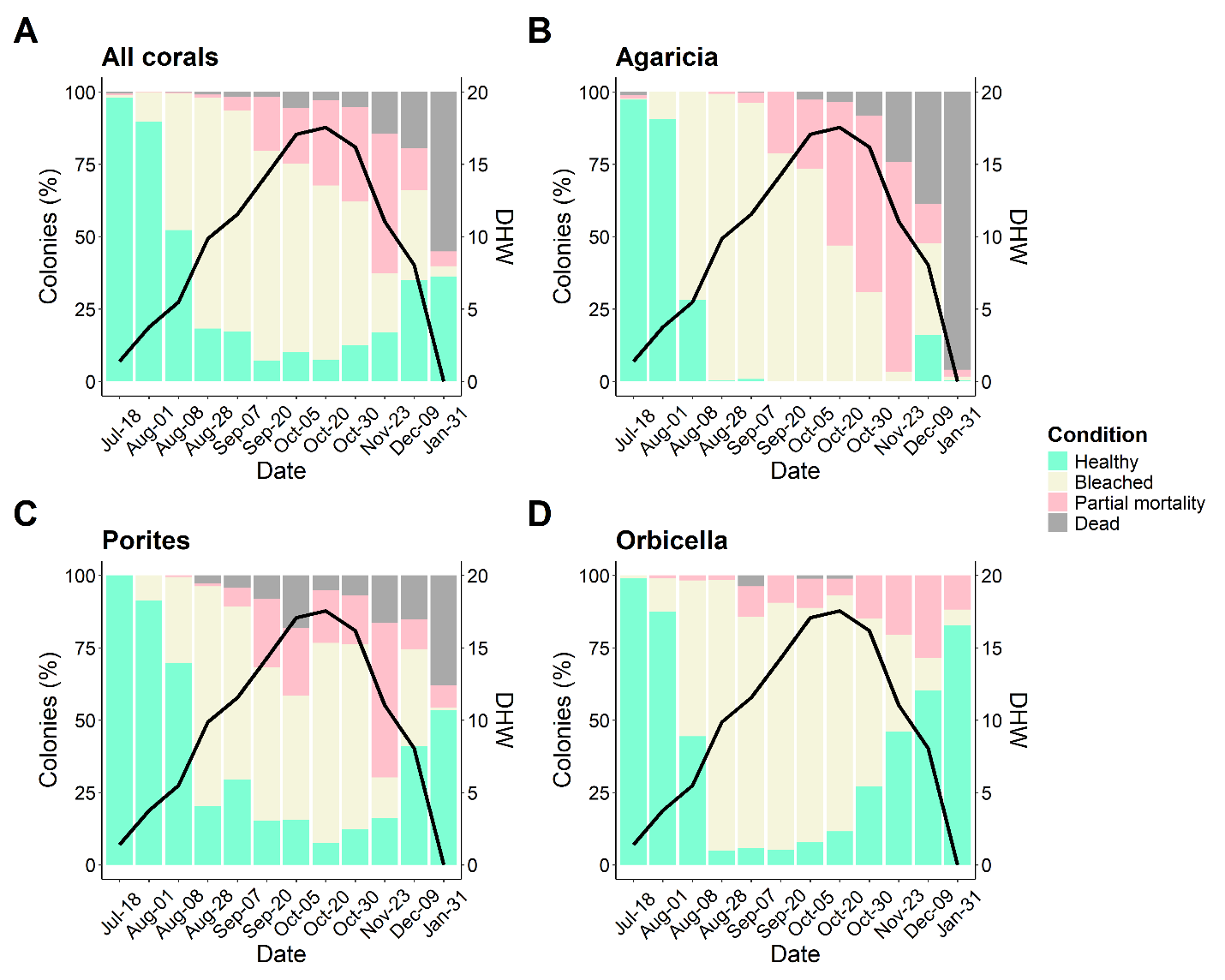


**Fig S1**. Overview of coral health conditions for (**A**) all corals, (**B**) *Agaricia spp*., (**C**) *Porites spp*., and (**D**) *Orbicella spp*. Black solid line shows degree heating weeks (DHW) throughout the survey period.
